# The New Medical Examiners Law

**Published:** 1890-07

**Authors:** 


### THE NEW MEDICAL EXAMINERS LAW.

The following is the text of the bill approved June 5, 1890, relating to the licensing of practitioners of medicine in the State of New York:

AN ACT TO ESTABLISH BOARDS OF MEDICAL EXAMINERS OF THE STATE OF NEW YORK FOR THE EXAMINATION AND LICENSING OF PRACTITIONERS OF MEDICINE AND SURGERY; AND TO FURTHER REGULATE THE PRACTICE OF MEDICINE AND SURGERY.

*The People of the State of New York, represented in Senate ana Assembly, do enact as follows:*

Section 1. From and after the first day of September, eighteen hundred and ninety-one, there shall be and continue to be three separate boards of Medical examiners for the State of New York, one representing the Medical Society of the State of New York, one representing the Homeopathic Medical Society of the State of New York, and one representing the Eclectic Medical Society of the State of New York. Each board shall consist of seven members, and each of said members shall serve for a term of three years, from the first day of September next after his appointment, with the exception of those first appointed, who shall serve as follows, viz.: Two of each board for one year, two of each board for two years, and three of each board for three years from the first day of September eighteen hundred and ninety-one. The power of appointment shall vest in the Board of Regents of the University of the State of New York, which shall appoint the members of said boards of examiners respectively from lists of nominees to be submitted by each of the said three medical societies, the number of nominees by each of said societies to equal or exceed twice the number of appointments so to be made from each of said societies. Each of said nominees shall be nominated by a majority vote at the annual meetings of the society with which said nominee may be in affiliation, and the names of persons so nominated shall be transmitted before the first day of July eighteen hundred and ninety-one to the said Board of Regents, under the seal of and signed by the president and secretary of the society so nominating. From these lists of nominees, respectively, said Board of Regents shall, prior to or during the month of July, eighteen hundred and ninety-one, appoint three separate boards of examiners, each board to be composed exclusively of members of the same medical society. In case of failure of any or all of said medical societies to submit nominees as aforesaid, said Board of Regents shall, prior to or during the month of July, eighteen hundred and ninety-one, appoint members in good standing of the corresponding society or societies entitled to nominate, without other restriction. Each one of said appointees, prior to appointment, shall furnish evidence of having received the degree of doctor of medicine in course from some legally incorporated medical college authorized to confer the same, and shall certify to said Board of Regents to having practiced medicine or surgery under the laws of this state for a period of not less than five years immediately prior to such appointment. The said Board of Regents shall fill vacancies by death or otherwise, for unexpired terms of said examiners from the respective lists of nominees submitted by the said medical societies ; and may remove any member of either of said boards for continued neglect of the duties required by this act, or on recommendation of the medical society of which said members may be in affiliation for unprofessional or dishonorable conduct. The Board of Regents shall, in their first appointments, designate the number of years for which each appointee shall serve. The appointments of successors to those members whose terms of office will expire on the first day of September of each year, shall be made by the Regents during or before the month of July of such year, upon the same conditions and requirements as hereinbefore specified with reference to the appointment of the three separate examining boards, each to be composed exclusively of members of the same medical school and society as are hereinbefore provided.

§ 2. Said boards shall be known by the name and style of Boards of Medical Examiners of the State of New York. Every person who shall be appointed to serve on either of said boards shall receive a certificate of appointment from the Regents of the University, and within thirty days after receiving such certificate shall take, subscribe and file in the office of the Secretary of State the oath prescribed by the twelfth article of the constitution of this state. Each of said boards shall be authorized to take testimony concerning all matters within its jurisdiction, and the presiding officer, for the time being of either of said boards, or of any of the committees thereof, may issue subpenas and administer oaths to witnesses. Each of said boards of examiners shall make and adopt all necessary rules, regulations and by-laws not inconsistent with the constitution and laws of this state or of the United States, whereby to perform the duties and transact the business required under the provisions of this act, said rules, regulations and by-laws to be subject to the approval of said Regents.

§ 3. From the income provided in this act the Regents may pay, not to exceed said income, all proper expenses incurred by its provisions; and if any surplus above said expenses shall remain at the end of any year, it shall be apportioned by said Regents among said examiners pro rata, according to the number of candidates examined by each.

§ 4. The first meeting of each of the examining boards respectively shall be held pursuant to a call issued by the secretary of the Board of Regents, within two months from the first day of September, eighteen hundred and ninety-one, suitable notice in the usual form being given to each of the members thereof, specifying the time and place of meeting. At the first meeting of each of the boards respectively, an organization shall be effected by the election, from their own membership, of a president and secretary. For the purpose of examining applicants for license, each of said boards of medical examiners shall hold one or more stated or special meetings in each year, pursuant to a call of the Board of Regents, due notice of which shall be made public, at such times and places as may be determined by the Board of Regents; but each examination shall be under the supervision of an examiner appointed by the Board of Regents, and who shall not be a member of any board of medical examiners. At said stated or special meetings a majority of the members of a board shall constitute a quorum thereof, but the examination may be conducted by a committee of one or more members of the board of examiners, duly authorized by such board.

§ 5. The several boards of medical examiners shall submit to the Board of Regents lists of examination questions for thorough examinations in anatomy, physiology and hygiene, chemistry, surgery, obstetrics, pathology and diagnosis, and therapeutics, including practice and materia medica; from the lists of questions so submitted the Board of Regents shall select the questions for each examination, and present the same to the candidates at each examination by an examiner appointed therefor by the Board of Regents, and such questions for each examination shall be so selected as to require the same standard of excellence from all candidates, except that in the department of therapeutics, practice and materia medica the questions shall be in harmony with the tenets of the school selected by the candidate.

§ 6. Said examinations shall be conducted in writing, in accordance with the rules and regulations prescribed by the Board of Regents, and shall embrace the subjects named in section five of this act. At the close of said examination, the examiner appointed by the Board of Regents having supervision thereof, shall forthwith deliver to the boards of medical examiners having charge of such examination, or to their duly authorized committee, the questions submitted to and the anwers of each applicant, and such boards of medical examiners, without unnecessary delay, shall transmit to the Regents of the University an official report, signed by the president, secretary and each acting member of said board of examiners, stating the examination average of each candidate in each branch, the general average, and the result of the examination, whether successful or unsuccessful. Said report shall embrace all the examination papers, questions and answers thereto. All the examination papers so returned shall be kept for reference and inspection among the public records of the University.

§ 7. On receiving from either of said boards of medical examiners such official report of the examination of any applicant for license, the said Regents shall issue to every applicant who shall have been returned as having successfully passed said examination, and who shall in their judgment be duly qualified therefor, a license to practice medicine and surgery in the State of New York. The Board of Regents shall require the same standard of qualifications from all candidates, except in the department of therapeutics, practice and materia medica, in which the standard shall be determined by each of the boards of medical examiners respectively. Every license to practice medicine or surgery, issued pursuant to this act, shall be subscribed by the chancellor and secretary of the University of the State of New York, by each medical examiner who reported the licentiate as having successfully passed the examinations, and also by those of the Regents who examined and approved the credentials of said licentiate upon the application for examination. It shall also have affixed to it, by the person authorized to affix the same, the seal of said University. Every such license shall be substantially in the following form:

"The Regents of the University of the State of New York. To all whom it may concern, greeting:

Be it known that A. B., on the . . . day of . . . . . A. D. . . having offered to us satisfactory proof that . . . . . was more than twenty-one years of age, and had received proper preliminary education; that . . . . . . had attended three full courses of medical instruction, the last course at . . . . . . . in . . . . . . . in the years of . . . . . , and had received from the . . . . . of . . . . . . . the degree of doctor of medicine; we thereupon gave a written order for the examination of said A. B. before one of the boards of medical examiners of the State of New York; that the said A. B. was fully examined before said board and found proficient and qualified to practice medicine and surgery by the examiners, whose signatures are hereunto attached. We, therefore, have granted to said A. B. this our license to practice medicine and surgery in the State of New York as a physician and surgeon, and have caused the names of the chancellor and secretary of our Board of Regents and said examiners to be subscribed, and the seal of the University to be affixed hereto, and have also caused this license to be recorded in book . . . of medical licenses, on page . . . . " Before said license shall be issued, it shall be recorded in a book to be kept in the office of said Regents, and the number of the book and the page therein containing said recorded copy shall be noted in the body of the license. Said records shall be open to public inspection, under proper restrictions as to their safe-keeping, and in all legal proceedings shall have the same weight as evidence that is given to the record of the conveyances of land.

§ 8. From and after the first day of September, eighteen hundred and ninety-one, any person not theretofore lawfully authorized to practice medicine and surgery in this State, and desiring to enter upon such practice, may deliver to the Regents of the University, upon the payment of twenty-five dollars into the treasury of the University of the State of New York, a written application for license, together with satisfactory proof that the applicant is more than twenty-one years of age, is of good moral character, has obtained a competent common school education, and has either received a diploma conferring the degree of doctor of medicine from some legally incorporated medical college in the United States, or a diploma or license conferring the full right to practice all the branches of medicine and surgery in some foreign country, and has also studied medicine three years, including three courses of lectures in different years, in some legally incorporated medical college or colleges prior to the granting of said diploma or foreign license; provided that two courses of medical lectures, both of which shall be either begun or completed within the same calendar year, shall not satisfy the above requirement. Such proof shall be made, if required, upon affidavit. Upon the making of said payment and proof, the Board of Regents, if satisfied with the same, shall direct the secretary thereof to issue to said applicant an order for examination by any one of said boards of medical examiners which said applicant may elect. In case of failure at any such examinations, the candidate, after the expiration of six months, and within one year, shall have the privilege of a second examination by the same board to which application was first made, without the payment of an additional fee. And it is further provided that applicants examined and licensed by State examining boards of other States, on payment of ten dollars to the University of this State, and on filing in the office of said Regents a copy of said license, certified by the affidavit of the president and secretary of such board, showing also that the standard of acquirements adopted by said State Examining Board is substantially the same as is provided by sections five and six of this act, shall, without further examination, receive from said Regents a license conferring on the holder thereof all the rights and privileges provided by sections eight and nine of this act.

§ 9. On and after the first day of September, eighteen hundred and ninety-one, no person not theretofore a legally authorized practitioner of medicine and surgery, under the laws of this State then in force, shall practice medicine or surgery in this State, unless that person shall have received from the Regents of the University, after examination and approval, as herein provided, a license to practice as a physician and surgeon, and unless said license shall have been registered as required under the provisions of chapter six hundred and forty-seven of the laws of eighteen hundred and eighty-seven, or, unless such person shall hold a license from a State examining and licensing board of another State, and shall have been licensed by the Board of Regents, as provided by this act.

§ 10. Nothing in this act shall be construed to interfere with or punish commissioned medical officers serving in the army or navy of the United States, or in the United States Marine hospital service while so commissioned, or any one while actually serving as a member of the resident medical staff of any legally incorporated hospital, or any legally qualified and registered dentist exclusively engaged in practicing the art of dentistry, or interfere with manufacturers of artificial eyes, limbs, or orthopedical instruments or trusses of any kind from fitting such instruments on persons in need thereof; or any lawfully qualified physicians and surgeons residing in other States or countries, meeting regular physicians and surgeons of this State in consultation, or any physician or surgeon residing on the border of a neighboring State and duly authorized under the laws thereof to practice medicine or surgery therein, whose practice extends into the limits of this State; providing that such practitioner shall not open an office or appoint a place to meet patients or receive calls within the limits of the State of New York; or physicians duly registered in one county of this State called to attend isolated cases in another county but not residing or habitually practicing therein.

§ 11. This act shall take effect immediately.

